# A new method of construction waste classification based on two-level fusion

**DOI:** 10.1371/journal.pone.0279472

**Published:** 2022-12-27

**Authors:** Lin Song, Huixuan Zhao, Zongfang Ma, Qi Song

**Affiliations:** 1 College of Information and Control Engineering, Xi’an University of Architecture and Technology, Xi’an, China; 2 Unmanned System Research Institute, Northwestern Polytechnical University, Xi’an, China; BMS Institute of Technology and Management, INDIA

## Abstract

The automatic sorting of construction waste (CW) is an essential procedure in the field of CW recycling due to its remarkable efficiency and safety. The classification of CW is the primary task that guides automatic and precise sorting. In our work, a new method of CW classification based on two-level fusion is proposed to promote classification performance. First, statistical histograms are used to obtain global hue information and local oriented gradients, which are called the hue histogram (HH) and histogram of oriented gradients (HOG), respectively. To fuse these visual features, a bag-of-visual-words (BoVW) method is applied to code HOG descriptors in a CW image as a vector, and this process is named B-HOG. Then, based on feature-level fusion, we define a new feature to combine HH and B-HOG, which represent the global and local visual characteristics of an object in a CW image. Furthermore, two base classifiers are used to learn the information from the color feature space and the new feature space. Based on decision-level fusion, we propose a joint decision-making model to combine the decisions from the two base classifiers for the final classification result. Finally, to verify the performance of the proposed method, we collect five types of CW images as the experimental data set and use these images to conduct experiments on three different base classifiers. Moreover, we compare this method with other extant methods. The results demonstrate that our method is effective and feasible.

## 1. Introduction

As a global issue, construction waste (CW) obstructs the sustainable development process of the construction industry. For instance, in the European Union, the construction sector generates over 500 million metric tons of CW per year, accounting for 50% of the waste produced in the EU [1]. China, as a rapidly developing country, is suffering from the issue of increasing CW. In China, researchers have found that CW accounts for 30%-40% of the total urban waste [2]. As urbanization accelerates and the population increases, the amount of CW will continue to increase. Formerly, CW was collected and stacked in a landfill. It occupies land, pollutes groundwater and contaminates air. In Nigeria, Modu *et al*. [3] considered recycling methods as an effective and sustainable strategy for solid waste management. Recycling waste materials not only provides economic benefits but also minimizes environmental issues. In China, according to the composition of CW, experts estimate that 95% of CW can be reused. However, due to the limited effect of CW classification, the utilization rate is less than 5% in complex real-life scenarios [4]. In harsh operating environments, manual sorting is accompanied by a number of issues regarding health, safety, efficiency, and expense. These problems reduce the propensity to recycle CW from landfills. As a significant procedure in CW recycling practices, the CW classification methods are rapidly being adjusted due to legislative and economical drivers [5].

With the evolution of technology, classification approaches based on deep learning have been used in various fields. Talo *et al*. [6] used ResNet-50 to classify histopathology images and obtained a better result. In order to improve the performance of image denoising, Zheng *et al*. [7] proposed a denoising CNN, which consists of a dilated block, a RepVGG block, a feature refinement block, and a single convolution. In addition, a pure transformer was applied directly to sequences of image patches and performed very well on image classification tasks [8]. Peter *et al*. [9] proposed a deep convolutional neural network to identify typical CW. However, the superior adaptability of deep learning models relies on large datasets. On small or medium-sized datasets, methods with handcrafted features may outperform deep learning methods. For example, Scale Invariant Feature Transform (SIFT) and Speeded-Up Robust Features (SURF) are integrated for image retrieval [10]. Because SIFT is robust to the changes in scale and rotation, SURF is robust to the changes in illumination. Furthermore, features play an important role in improving classification accuracy [11]. Therefore, researchers combined BoVW with feature descriptors to improve the performance. Abouzahir *et al*. [12] used BoVW to represent the HOG descriptors in weed detection. The result demonstrated that using BoVW can improve the performance of HOG. Rao *et al*. [13] used the BoVW technique to label the SIFT features extracted from X-ray images as fractured or non-fractured. Local binary patterns (LBP) and the BoVW model were combined for detecting soybean diseases [14]. Aslan *et al*. [15] used the BoVW technique to reinforce the SURF features in human activity recognition.

Recently, with the development of sensing technology, many researchers have been working on the automatic classification of CW through crushing, magnetic separation, computer vision, and other processes. Among these methods, computer vision stands out for its rapidity, low cost, and applicability in different scenes. For example, Setiawan *et al*. [16] designed a method to identify organic garbage. Lee *et al*. [17] proposed a method based on machine learning to separate reinforcing bar areas from the background. Using image processing technology, Zhao *et al*. [18] built hardware and software systems to identify steel bars. In summary, these methods can only identify one specific material. In industry, the applicability of these methods is limited because various CW materials are collected in landfills. To automatically detect CW, Rashidi *et al*. [19] extracted the color histogram and the dominant edge histogram of three building materials and evaluated the performance of the proposed method with three different machine learning techniques (the multilayer perceptron, radial basis function, and support vector machine (SVM) methods). Xiao *et al*. [20, 21] used near-infrared hyperspectral technology to capture CW images and extract the characteristic reflectivity. In this study, according to the requirements of the management department, CW is divided into concrete, brick, plastic, foam, and wood. We use an industrial camera to capture the CW images. Compared with near-infrared hyperspectral cameras, industrial cameras can reduce costs for users. To obtain complementary information, the features are extracted from the global and local views of CW images. An efficient classification model based on feature-level fusion and decision-level fusion is proposed to improve the performance of CW classification.

A preliminary version of this work was presented in CCC2022. In this paper, we substantially revised and extended the original paper. The main extensions include using color features to divide CW into salient objects (*i*.*e*., bricks and wood) and other objects, and building a joint decision model based on a fusion mechanism. These extensions substantially improve the classification performance. The remainder of this paper is organized as follows. The capturing system and the CW classification framework based on two-level fusion are described in section 2. In section 3, we evaluate the performance of the proposed method and compare it with others. Finally, we summarize our work and give some remarks on future research directions in section 4. The automatic sorting of CW can be realized with our method.

## 2. Materials and methods

### 2.1 Capturing system

**Fig 1:**
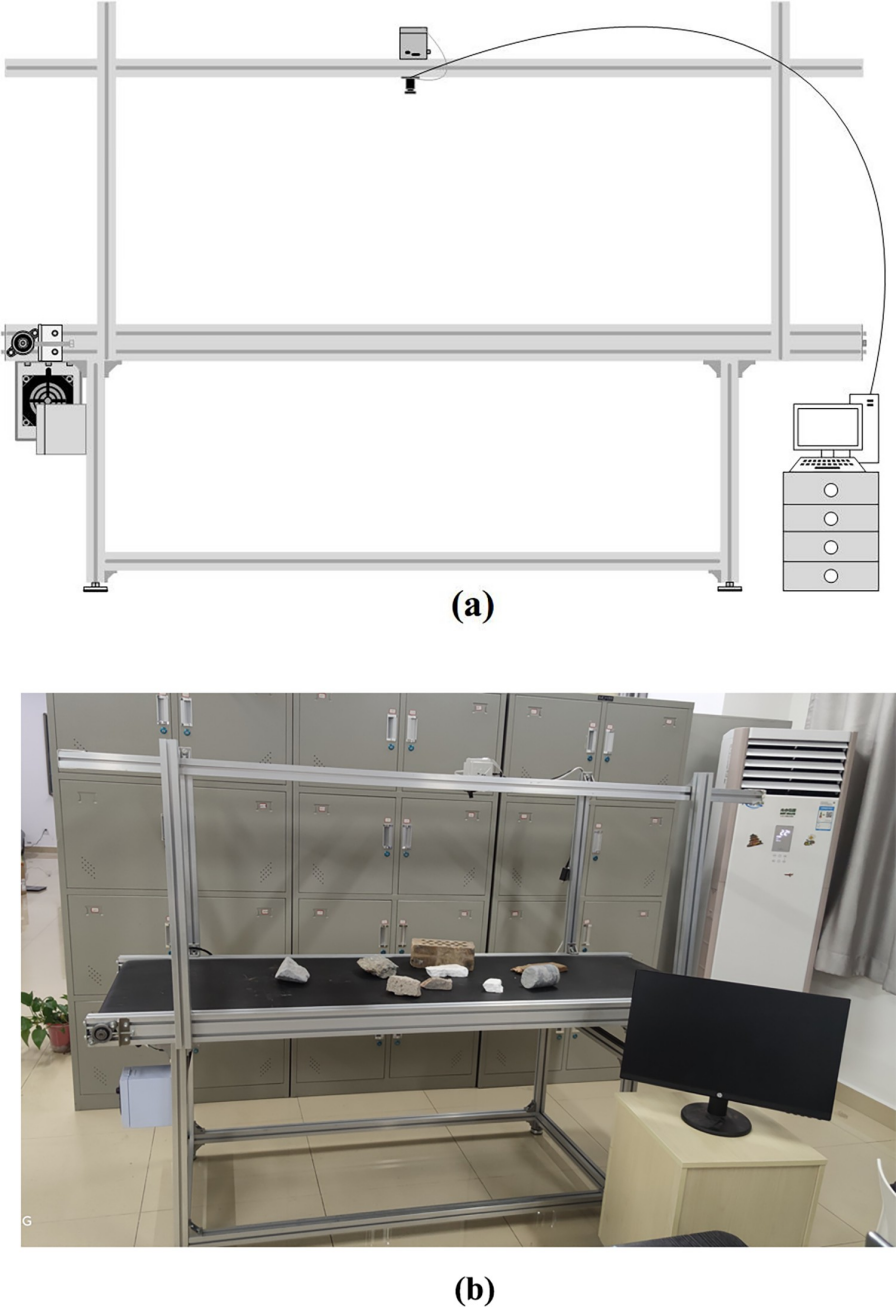
Capturing system. (a) System structure. (b) Real system.

For image acquisition, we use an industrial camera to capture images. The camera is fixed on a metal frame located directly above the belt. To ensure that the objects on the belt can be captured, the height of the metal frame is variable. It can avoid confusing extraneous features, which will influence the experimental results. The data acquisition system is shown in **Fig 1**. The speed of the conveyer belt varies with the needs of users. The governor controls the speed of the motor. In this experiment, the conveyer belt runs at 100 mm per second. The industrial camera frame rate is 10 fps. The screen is used to display the CW images and experimental results.

### 2.2 Experimental materials

**Fig 2:**
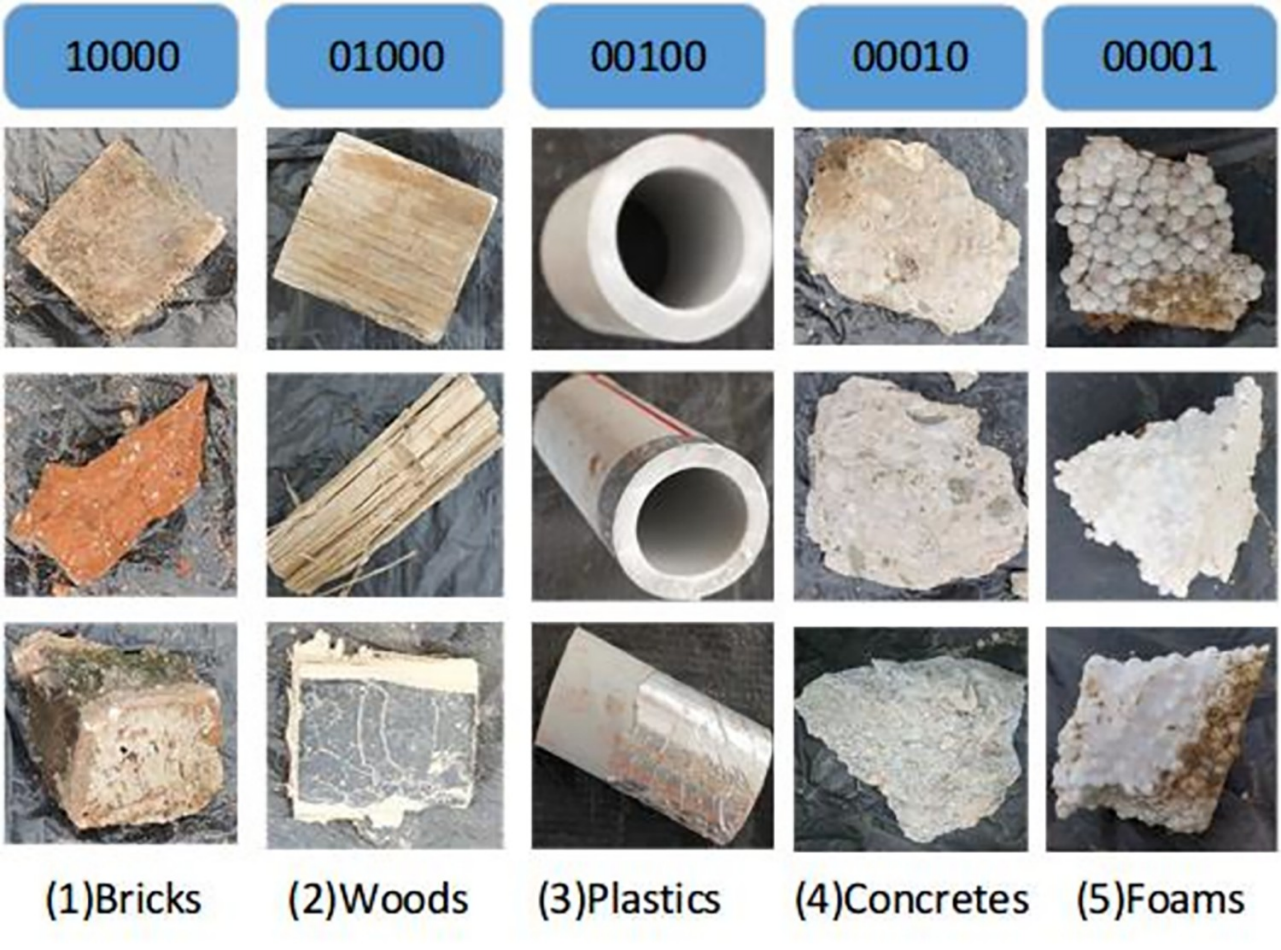
Five types of CW images and labels. The first row is the label, and the others are images.

In this study, we mainly classify materials, such as concretes, bricks, plastics, foams, and woods, provided by a construction management department. We capture 125 pictures of each class by using the capturing system, and 100 samples of each type are used as training data. These samples were picked from the waste collection site and were not cleaned to ensure that each sample contained pollution. These images are labeled as an array of 0 and 1 values. Each label array has five elements. If the image is class M, the corresponding element is 1, and the others are 0. The sample images and corresponding labels of the five types of CW materials are shown in **Fig 2**. The appearance of the concrete is rough and white. Plastic, as a common CW, has a smooth surface. Foam can be different colors because its surface often attaches to other materials. Wood has a rectangular appearance. The color of bricks depends on their constituents. They are often orange or red. These materials are used in different construction stages.

### 2.3 Extracting visual features

#### (1) The global hue features

Color is a comparatively robust visual feature that is invariant to object scale and position [22]. Similarly, color information is a main feature that allows humans to recognize objects. Since the hue-saturation-value (HSV) model is consistent with the characteristics of the human visual system, the HSV feature is one of the main features for pattern recognition [23, 24]. The HSV color space can be described as a conical geometry with three parameters, *i*.*e*., hue, saturation, and value [25]. In this method, we use the information from the hue channel to classify CW. We convert the image captured by the industrial camera from RGB space to HSV space. Next, we extract the hue information from the global perspective of the image. Finally, HH is used to describe the color information of the image. The algorithm flow for extracting HH is shown in Algorithm 1.

**Algorithm 1**: Extract HH

**Input**: Digital images

**Output**: Hue histogram (HH) of the images

Step 1: Convert the image from RGB color space to HSV color space.

Step 2: For each image, extract the hue information in the HSV color space.

Step 3: Use the histograms to represent the hue information of the CW images.

#### (2) The locally oriented gradient features

The histogram of oriented gradients (HOG) is a good feature for describing the shape and texture information of an object [26]. The process of computing HOG starts by dividing an image into cells and grouping the cells into blocks [23]. In each block, we calculate the gradient magnitude and gradient orientation, which are shown in **Eqs (1)** and **(2)**.

$$M=\sqrt{{m_{y}(x,y)}^{2}+{m_{x}(x,y)}^{2}}$$ (1)

$$\theta =\tan^{-1}\frac{m_{y}(x,y)}{m_{x}(x,y)}$$ (2)

where m_y_ and m_x_ are the vertical and horizontal gradients counted by the 1D filter. M and *θ* represent the gradient magnitude and gradient orientation of pixel (*x*,*y*), respectively. The detailed processing is referenced in [27], and we name it EXTRACTHOG (∙).

The traditional HOG model of an image is constructed by HOG descriptors of blocks. It usually does not provide good classification performance due to redundant information. To reduce the dimensions and maintain the discrimination of HOG, a BoVW method is used to code HOG models of all blocks in an image. We name the new feature $F_{B-HOG}$. Assume there are *N* classes of CW images and each class has *M* images for training, denoted as $\left\{I_{j}^{\left(i\right)},i=1,\ldots ,N,j=1,\ldots ,M\right\}$. The procedure of extracting $F_{B-HOG}$ is shown in **Algorithms 2 and 3.**

**Algorithm 2**: Build a bag on training images 

**Input**: Training images $\left\{I_{j}^{\left(i\right)},i=1,\ldots ,N,j=1,\ldots ,M\right\}$, parameters *K*,*L*

**Output**: Bag of visual words B

**Initial**: the bag of the *i*^*th*^ class ***B***^(*i*)^ = [], the whole bag B=[]

    **For** *i* = 1:*N*

        **For** *j* = 1:*M*

            Divide $I_{j}^{\left(i\right)}$ into *L* blocks $S_{l}$, *l* = 1,…,*L*

            **For** *l*←1 to *L* do:

                ***b***_*jl*_←EXTRACTHOG $\left(S_{l}\right)$

            **End**

            $B^{\left(i\right)}\leftarrow \{B^{\left(i\right)},b_{jl}\}$

        **End**

        Using K-means on {***B***^(*i*)^} to obtain *K* centers $V^{\left(i\right)}=\{v_{n},n=1,\ldots ,K\}$

        $B\leftarrow \{B,V^{\left(i\right)}\}$

    **End**

**Algorithm 3**: Coding on the whole HOG descriptors 

**Input**: Bag of visual words B, parameters *K*,*L*, the target image ***I***_*t*_

**Output**: Coding vector $F_{B-HOG}$

**Initial**: $F_{B-HOG}$ ←zeros(1, *K*×*N*)

Step 1: Divide ***I***_*t*_ into *L* blocks.

Step 2: **For** *l*←1 to *L* do:

                **b**_*l*_←EXTRACTHOG $\left(I_{t}^{l}\right)$

                $\hat{n}={min}_{n\in \{1,\ldots ,K\times N\}}{\left\|b_{l}-v_{n}\right\|}_{2}$

                $F_{B-HOG}(1,\hat{n})\leftarrow F_{B-HOG}(1,\hat{n})+1$

        **End for**

Step 3:$F_{B-HOG}=F_{B-HOG}/{\left\|F_{B-HOG}\right\|}_{1}$

Step 4: Return $F_{B-HOG}$

### 2.4 Feature-level fusion

**Fig 3:**
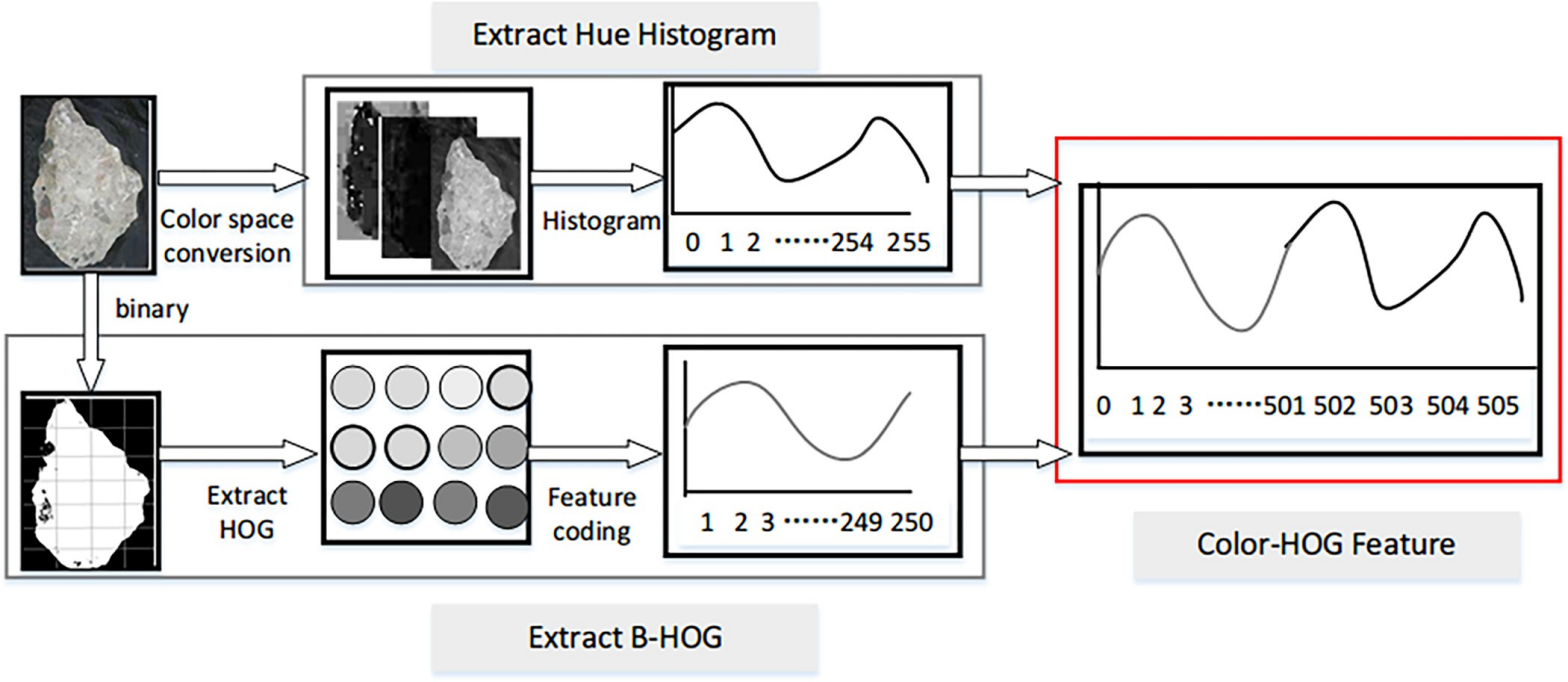
Building a new Color-HOG feature based on feature-level fusion.

As mentioned before, the color feature and HOG descriptors are extracted from multiple views. They can provide comprehensive information for CW classification. To combine the information from multiple views, we construct a new feature based on feature-level fusion and name it the Color-HOG feature. The overall flow is illustrated in **Fig 3**. For one sample image I, we convert it to HSV color space and extract the hue information from the HSV model. Then, the HOG descriptors are extracted from the local perspective of the image and coded by the BoVW method. Finally, the new Color-HOG feature $F_{CH}$ can be obtained through **Eq (3)**.

$$F_{CH}=[{n_{1}\times F}_{HH};n_{2}{\times F}_{B-HOG}]$$ (3)

where $F_{HH}$ and $F_{B-HOG}$ indicate the color feature and the coded HOG descriptors in image I, respectively. $n_{1}$ and $n_{2}$ are the weights to balance $F_{HH∙I}$ and $F_{B-HOG∙I}$. In this experiment, $n_{1}$ and $n_{2}$ are set to 1.

### 2.5 Decision-level fusion

**Fig 4:**
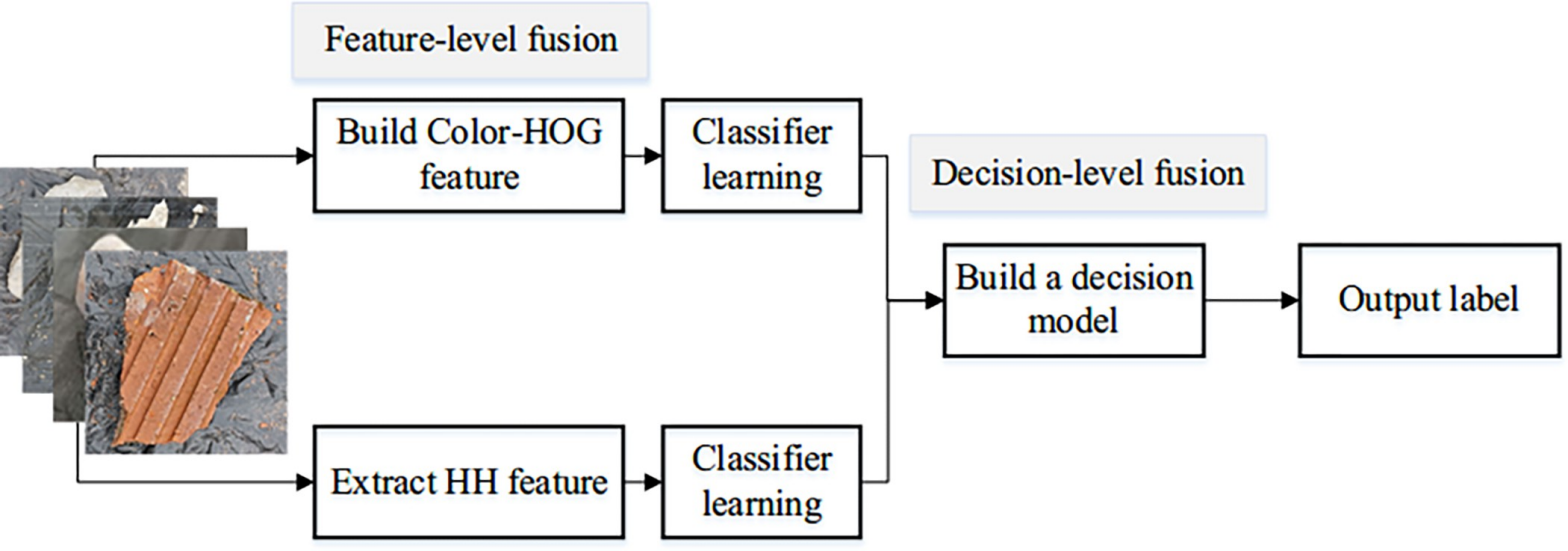
Overview of the CW classification method.

It is observed that bricks and woods have more color saliency than the other materials. This interesting phenomenon can help to distinguish salient materials from others. Therefore, two base classifiers are used to learn the information from the HH feature space and the Color-HOG feature space. We designed a joint decision model to combine the decisions from the feature spaces. The flowchart of CW classification is shown in **Fig 4**.

Inspired by the fusion mechanism, the new joint decision-making model is illustrated in **Eq (4)**. Let $P_{H}$ and $P_{CH}$ be the outputs of base classifiers, which are learned from the HH feature space and Color-HOG feature space, respectively. It is noteworthy that $P_{H}$ is a vector containing two probability values. The probabilities are used to determine whether the object belongs to the categories with salient color. $P_{CH}$ is a vector containing five elements, and each element represents the probability that the sample belongs to the category of CW. ℝ is a transition matrix. $P_{HCH}$ is the result of the decision-making model.

$$P_{HCH}=\omega_{1}\times R^{T}\times P_{H}+\omega_{2}\times P_{CH},\;s.t.\omega_{1}+\omega_{2}=1$$ (4)

R=[0η10η2η30η40η50],∑i=12ηi=1,∑i=35ηi=1 (5)

where *ω*_1_ and *ω*_2_ are the fusion weights of $P_{H}$ and $P_{CH}$. When *ω*_1_ = 0, the evidence from the Color-HOG feature space is completely credible. In this case, we classify CW based on feature-level fusion and name the method as the feature-level fusion-based CW classification (FLF). When *ω*_1_ = 1, the decision model indicates that only the HH feature is used to classify CW. In this case, since the HH feature can only effectively distinguish salient objects (*i*.*e*., bricks and woods) from the other materials, we cannot obtain an explicit label. For *ω*_1_∈(0,1), the CW classification method is based on feature-level fusion and decision-level fusion. We denote it as the two-level fusion-based CW classification method (TLF). It can be seen that FLF is a special case of TLF. In the next subsection, we investigate the influence of parameter *ω*_1_ tuning on the TLF algorithm.

## 3. Results and discussions

### 3.1 Salient features

**Fig 5:**
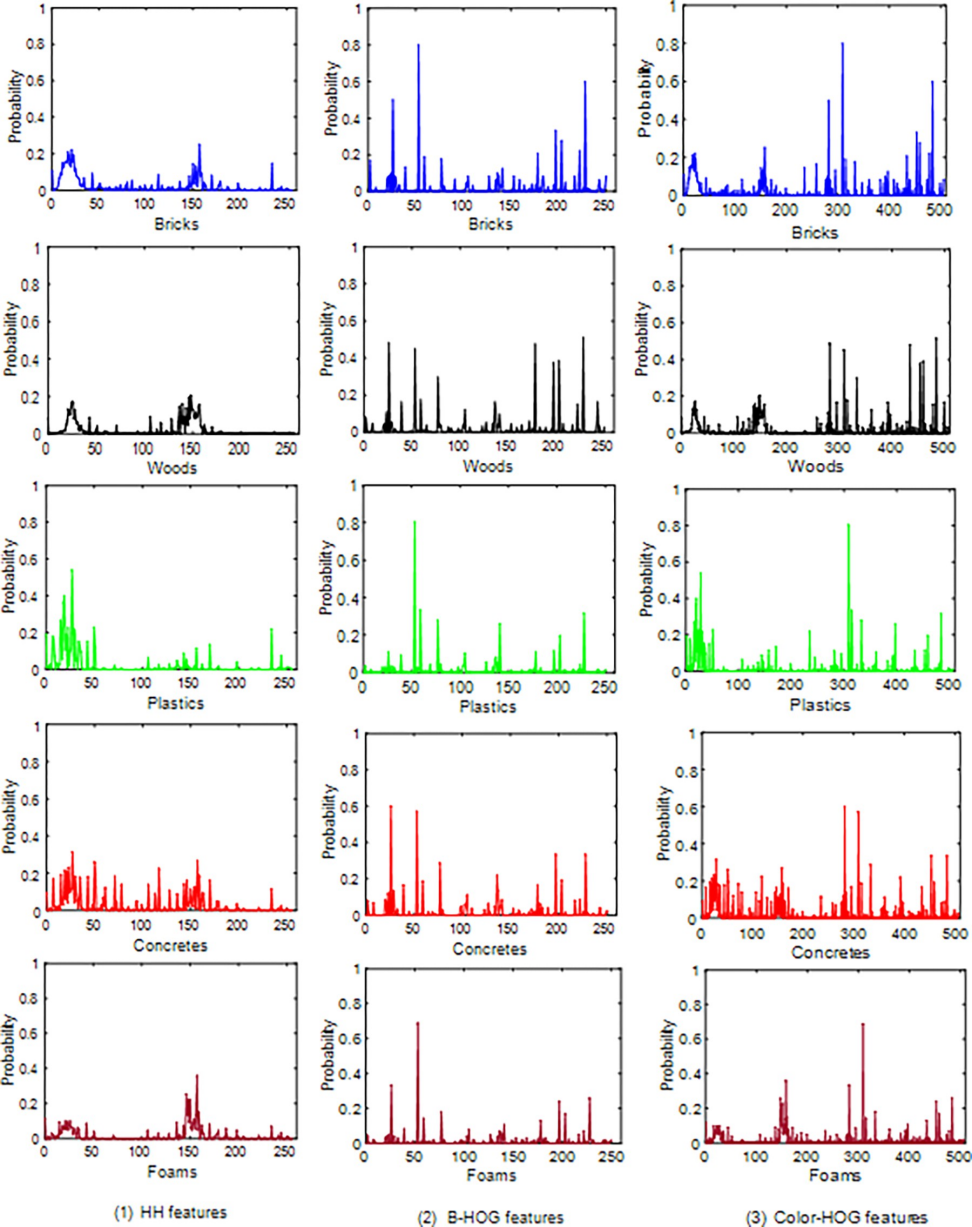
The envelopes of the salient features of the brick, wood, plastic, concrete, and foam materials. (1) HH features, (2) B-HOG features, and (3) Color-HOG features.

The envelopes of HH features, B-HOG features, and Color-HOG features of 5 CW categories are displayed in **Fig 5.** The HH features of the CW images are shown in the first rank. We conclude that the probability curve of each category has two peaks, but the information distributions are different. The first peaks of the brick and wood materials are thin. However, the peaks of the plastic, concrete, and foam materials are wide. Therefore, HH features can be used to distinguish salient objects (*i*.*e*., bricks and woods) from others and as the basis for CW classification. The B-HOG features of 5 CW categories are shown in the second rank of **Fig 5**. A bag-of-visual-words about HOG descriptors is built through the K-means algorithm. A histogram of the bag-of-visual-words is used to describe the local information of the CW images. The envelopes of the histograms show that the B-HOG features can be regarded as the basis for CW classification. Inspired by the fusion mechanism, the HH features and B-HOG features are connected to form Color-HOG features, which is shown in the third rank of **Fig 5**.

### 3.2 Effects of parameter tuning

The number of visual vocabularies is a significant parameter that affects the performance of the TFL method. We use the K-means clustering method to construct different sizes of BoVW and determine the optimal parameter. The accuracies of the TLF method with different sizes of visual vocabularies are shown in **Table 1**. Using a small number of visual vocabularies, different significant information may be combined into one cluster. As the size of the visual vocabulary increases, more CW details can be obtained, but a large vocabulary tends to overfit. The highest average accuracy of 96.32% is obtained on the BoVW with a size of 250. Therefore, the number of visual words in the proposed method is set at 250.

**Table 1:** Average accuracy of the proposed method with different sizes of vocabulary.

Vocabulary size	100	150	200	250	300	350	500	700
Accuracy	95.04%	96.00%	95.84%	**96.32%**	94.56%	95.52%	95.20%	95.36%

**Fig 6:**
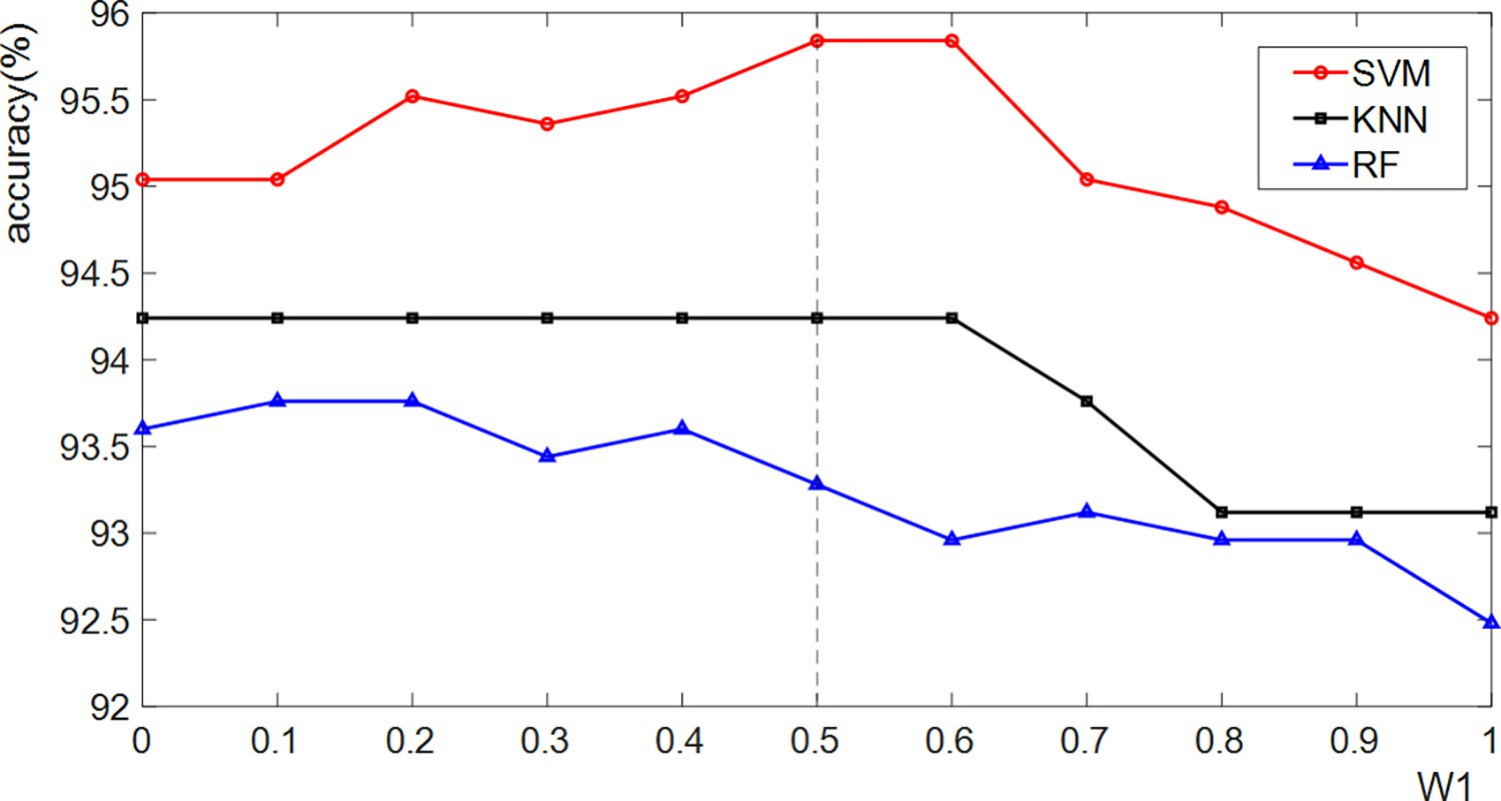
The influence of *ω*_1_ tuning on the joint decision-making model.

In the joint decision model, *ω*_1_ and *ω*_2_ represent the fusion weights of $P_{H}$ and $P_{CH}$, which satisfy *ω*_2_+*ω*_1_ = 1. To determine the optimal fusion weights, we conduct extensive experiments with different *ω*_1_. For *ω*_1_ = 1, only color features are used to classify CW, and this is described in **section 2.4**. In this instance, CW can be divided into salient objects and other materials. For ω_1_∈[0,1], the average accuracy curves of 5 CW categories are shown in **Fig 6**. When *ω*_1_<0.5, the evidence from the Color-HOG feature space plays a leading role. During this phase, the accuracy curves indicate that the classification accuracy increases as *ω*_1_ increases. For *ω*_1_>0.5, the evidence from the color feature space plays a dominant role, and the curves show a downward trend. This indirectly demonstrates that the features from the Color-HOG feature space and HH feature space are equally important. In our work, we recommend taking *ω*_1_ = 0.5 as a default value.

### 3.3 Evaluation of classification performance

**Fig 7:**
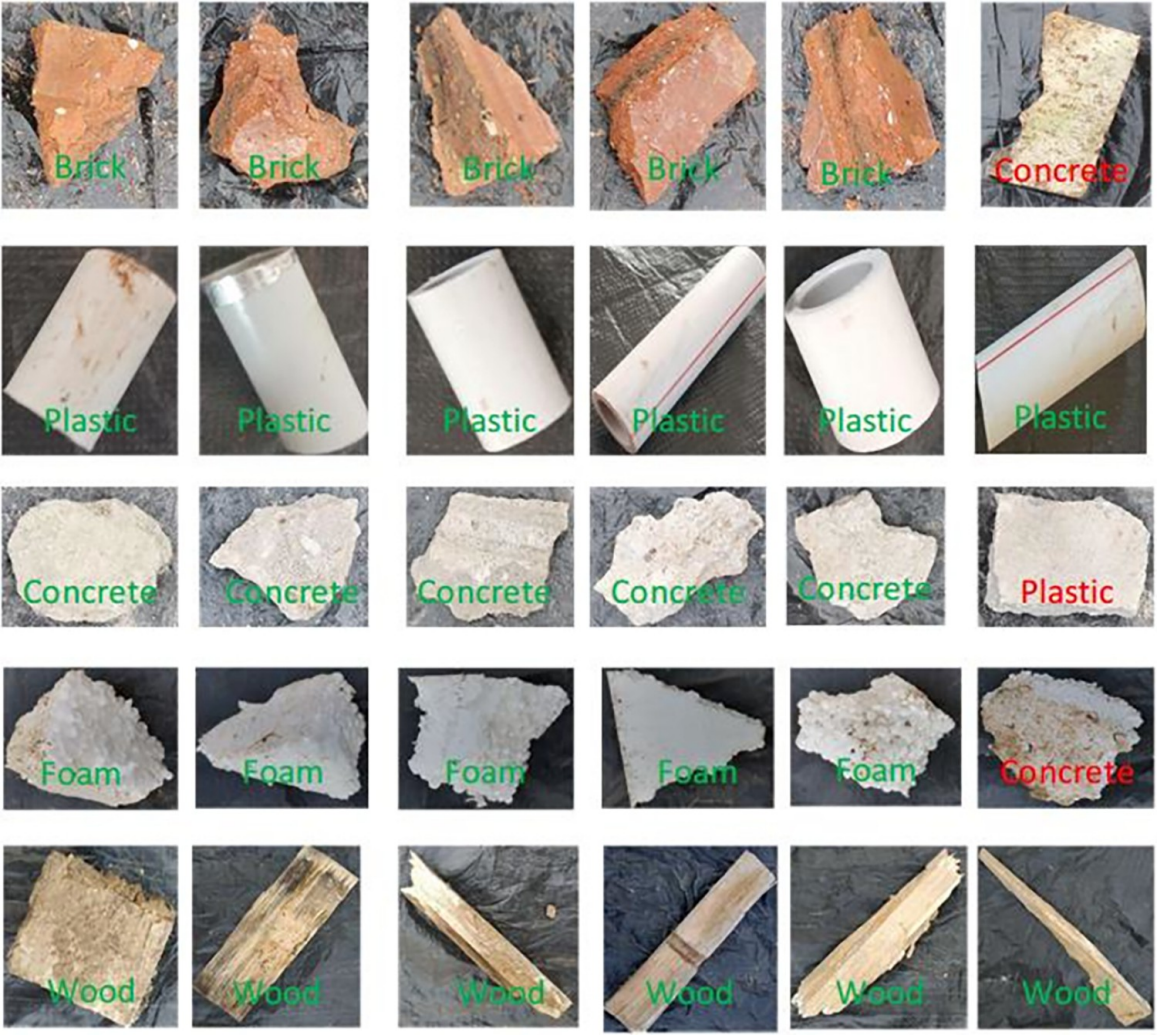
Example classification results of the TLF method based on the SVM classifier.

**Fig 8:**
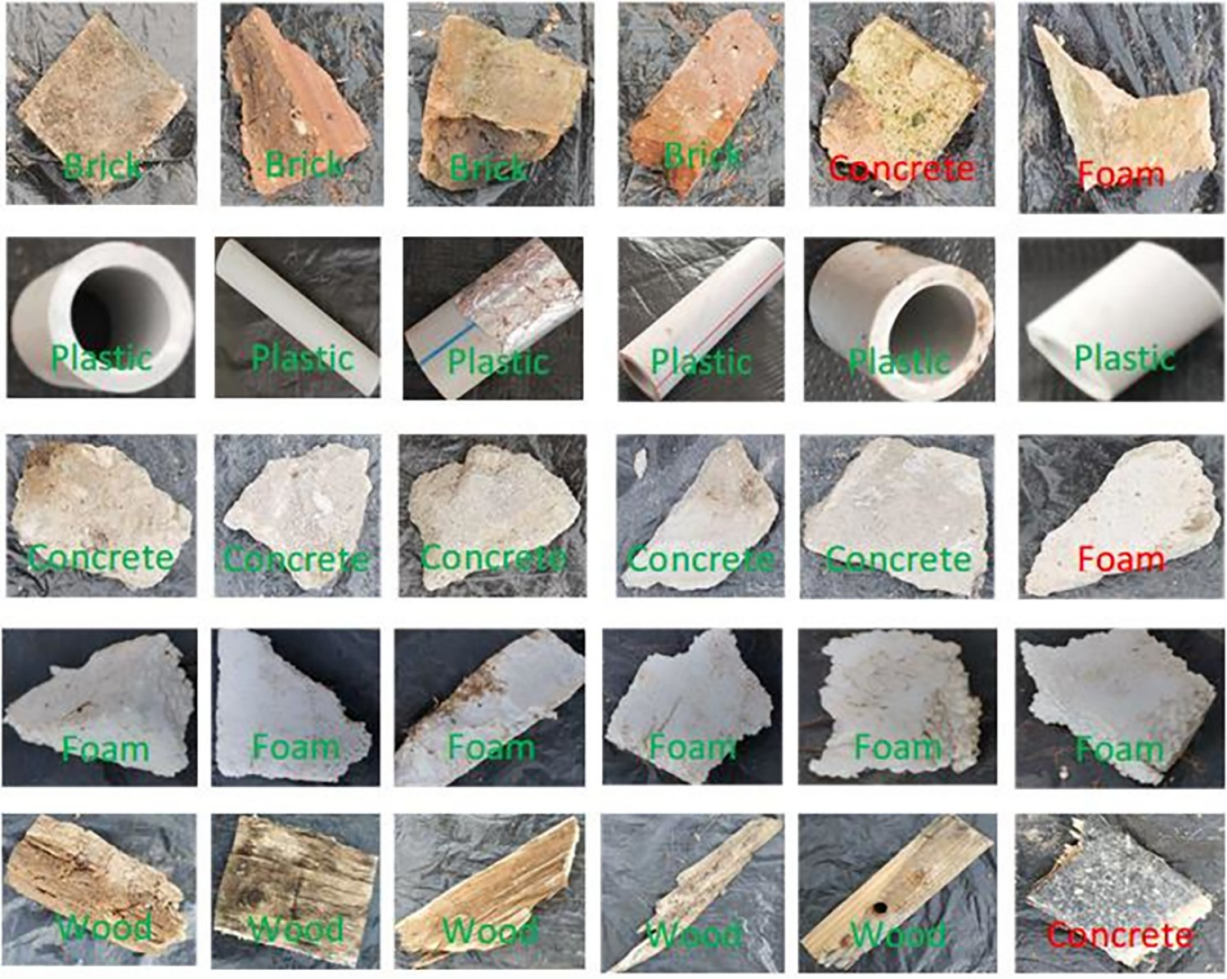
Example classification results of the FLF method based on the SVM classifier.

In this section, the CW images described in **section 2.2** are used to assess the performance of the proposed method. Five-fold cross validation is applied on all image sets. Confusion matrices are used to represent the performance of the proposed algorithm. The results of the proposed method on three base classifiers are shown in **Tables 2–4.** The base classifiers include the SVM [28], *K*-nearest neighbor (KNN) [29], and random forest (RF) [30] methods. The recall of plastics in the FLF method and TLF method based on the SVM classifier is 99.2%, which is higher than that of bricks, concretes, foams, and woods. **Table 3** shows that the recall of plastic is the highest, which is 98.4%. **Table 4** indicates that in the TLF method, the recall of bricks is higher than that of plastics. Generally speaking, the recall values of most materials are higher than 90%. But, based on the RF classifier, the recall of concrete and foam is 89.6% and 87.2%, respectively. However, if we replace the base classifier, the recall of concretes and foams can be improved. In other words, our proposed methods still show superior performance. In addition, precision is also used to evaluate the FLF and TLF methods. **Table 2** shows that the precision of the plastics in the FLF method and TLF method is 98.4% and 99.2%, respectively, which is higher than that of the other materials. Based on the KNN classifier, the precision of plastics is the highest, which is 98.4%. Although **Table 4** indicates that the results of concretes and foams are slightly confused, the average accuracies of the FLF method and TLF method are 92.8% and 93%, respectively. This may be related to the performance of the base classifiers. Overall, we can see that our proposed CW classification method achieves good performance. Some of the results are visualized in **Figs 7** and **8**. The correct classification results are marked in green, and the incorrect classification results are marked in red.

**Table 2:** Confusion matrix of CW classification based on SVM.

SVM classifier		Truth class					
		Bricks	Plastics	Concretes	Foams	Woods	Precision
**FLF**	Bricks	**118**	0	0	1	3	96.7%
	Plastics	0	**124**	2	0	0	98.4%
	Concretes	3	0	**115**	2	3	93.5%
	Foams	4	0	3	**120**	1	93.8%
	Woods	0	1	5	2	**118**	93.6%
	**Recall**	94.4%	99.2%	92%	96%	94.4%	**95.2%**
**TLF**	Bricks	**123**	0	1	0	2	97.6%
	Plastics	0	**124**	1	0	0	99.2%
	Concretes	2	0	**117**	3	3	93.6%
	Foams	0	0	2	**120**	2	96.8%
	Woods	0	1	4	2	**118**	94.4%
	**Recall**	98.4%	99.2%	93.6%	96%	94.4%	**96.3%**

**Table 3:** Confusion matrix of CW classification based on KNN.

KNN classifier		Truth class					
		Bricks	Plastics	Concretes	Foams	Woods	Precision
**FLF**	Bricks	**117**	0	2	0	3	95.9%
	Plastics	0	**123**	2	0	0	98.4%
	Concretes	7	1	**113**	2	0	91.9%
	Foams	1	1	4	**121**	6	91.0%
	Woods	0	0	4	2	**116**	95.1%
	**Recall**	93.6%	98.4%	90.4%	96.8%	92.8%	**94.4%**
**TLF**	Bricks	**117**	0	2	0	3	95.9%
	Plastics	0	**123**	2	0	0	98.4%
	Concretes	7	1	**113**	2	0	91.9%
	Foams	1	1	4	**121**	6	91.0%
	Woods	0	0	4	2	**116**	95.1%
	**Recall**	93.6%	98.4%	90.4%	96.8%	92.8%	**94.4%**

**Table 4:** Confusion matrix of CW classification based on RF.

RF classifier		Truth class					
		Bricks	Plastics	Concretes	Foams	Woods	Precision
**FLF**	Bricks	**113**	5	0	3	2	91.9%
	Plastics	4	**119**	5	0	0	93.0%
	Concretes	6	0	**115**	8	2	87.8%
	Foams	1	0	3	**113**	1	95.8%
	Woods	1	1	2	1	**120**	96.0%
	**Recall**	90.4%	95.2%	92%	90.4%	96%	**92.8%**
**TLF**	Bricks	**120**	5	0	5	2	90.9%
	Plastics	1	**119**	5	0	0	95.2%
	Concretes	2	0	**112**	8	2	90.3%
	Foams	0	0	3	**109**	0	97.3%
	Woods	2	1	5	3	**121**	91.7%
	**Recall**	96%	95.2%	89.6%	87.2%	96.8%	**93.0%**

### 3.4 Testing in various conditions

**Fig 9:**
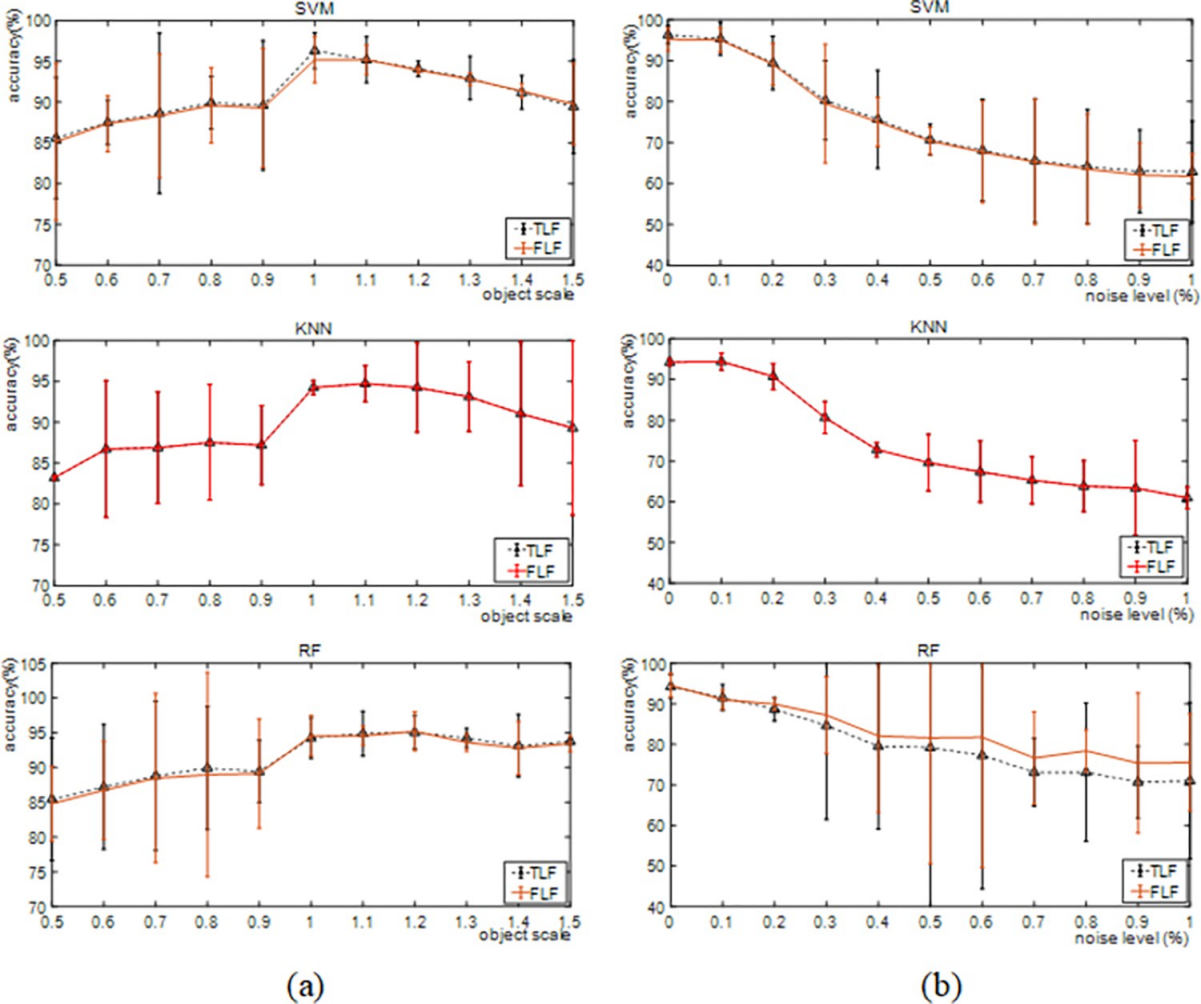
The robustness of the proposed methods, (a) the effect of scale changes, and (b) the effect of noise intensity.

In industry, the environment of the construction jobsite is variable. The shaking of the capturing system produces noise. In addition, because CW contains demolished materials, the sizes of the objects may vary. To verify the robustness of our proposed approaches, we test the performance of the TLF and FLF methods with different levels of noise and scales. The results are reported in **Fig 9**. As the scale changes, the accuracy curves show a slight downward trend. However, when the size of the CW image changes from a scale of 0.5 to 1.5, the overall accuracy is higher than 80%. With an increase in noise, the accuracy curves show a downward trend. When the noise intensity is lower than 0.3%, the classification accuracy of the proposed methods is higher than 80%. However, at a construction jobsite, the types of noise vary. Generally, the proposed methods have better performance.

### 3.5 Comparison of classification performance

To verify the effectiveness of the new Color-HOG feature in our proposed approach, we conduct comparison experiments with other traditional features, *i*.*e*., HOG [31], LBP [32], SURF [33], and SIFT [34]. In the comparison experiments, the traditional features are also coded by visual words. As shown in **Table 5**, based on the SVM classifier, the average accuracies of LBP-BOW [14], SURF-BOW [15], SIFT-BOW [13], HOG-BOW [12], and Gabor Wavelets [19] are 71.36%, 83.36%, 83.84%, 59.04%, and 94.72%, respectively. Moreover, **Tables 5–7** show that the FLF method and TLF method have more than 10% improvement compared with other methods except Gabor Wavelets. Unfortunately, on all tests, our proposed methods don’t always have the best results, such as the Gabor Wavelets method, which has the best results with the SVM classifier on Test2 datasets. However, if we replace the base classifier, our proposed methods can achieve the desired results. Therefore, the above phenomenon may relate to the potential of the base classifiers. Generally speaking, the average accuracy of our proposed method is better than that of the Gabor Wavelets method. In other words, our proposed methods still show competitive ability.

**Table 5:** Comparison with other classic features based on SVM classifier.

SVM classifier	Test1(%)	Test2(%)	Test3(%)	Test4(%)	Test5(%)	Average (%)
LBP-BOW [14]	76.0	72.8	75.2	62.4	70.4	71.36±5.47
SURF-BOW [15]	85.6	84.0	85.6	79.2	82.4	83.36±2.68
SIFT-BOW [13]	88.0	83.2	84.8	82.4	81.6	83.84±2.53
HOG-BOW [12]	59.2	60.0	62.4	59.2	54.4	59.04±2.91
Gabor Wavelets [19]	96.5	**96.3**	92.8	95.2	92.8	94.72±1.82
**FLF**	96.8	94.4	**97.6**	93.6	93.6	95.20±1.88
**TLF**	**98.4**	94.4	**97.6**	**96.0**	**95.2**	**96.32**±**1.66**

**Table 6:** Comparison with other classic features based on KNN classifier.

KNN classifier	Test1(%)	Test2(%)	Test3(%)	Test4(%)	Test5(%)	Average (%)
LBP-BOW [14]	68.8	64.0	64.0	57.6	60.8	63.04±4.17
SURF-BOW [15]	77.6	76.0	84.0	76.8	76.0	78.08±3.38
SIFT-BOW [13]	83.2	77.6	80.8	80.8	79.2	80.32±2.09
HOG-BOW [12]	52.8	59.2	61.6	54.4	60.8	57.76±3.94
Gabor Wavelets [19]	**95.4**	92.2	94.6	**95.2**	91.4	93.76±1.84
**FLF**	95.2	**92.8**	**96.0**	94.4	**93.6**	**94.40**±**1.26**
**TLF**	95.2	**92.8**	**96.0**	94.4	**93.6**	**94.40**±**1.26**

**Table 7:** Comparison with other classic features based on KNN classifier.

RF classifier	Test1(%)	Test2(%)	Test3(%)	Test4(%)	Test5(%)	Average (%)
LBP-BOW [14]	76.8	74.4	76.0	72.8	72.0	74.4±2.03
SURF-BOW [15]	85.6	82.4	84.0	78.4	85.6	83.2±2.99
SIFT-BOW [13]	87.2	82.4	81.5	80.2	85.7	82.56±3.22
HOG-BOW [12]	68.0	62.4	68.0	66.4	60.8	65.12±3.32
Gabor Wavelets [19]	91.6	91.6	95.4	93.2	90.8	92.52±1.83
**FLF**	**92.8**	91.2	95.2	**96.0**	89.6	92.96±2.68
**TLF**	**92.8**	**92.0**	**96.0**	94.4	**92.0**	**93.44**±**1.73**

In addition to the above-mentioned classic models based on handcrafted features, deep learning models have also become a trend. VGG-16 [9], ResNet-50 [6], and the Vision Transformer network (ViT) [8] are three classic deep learning models used for comparison with the TLF method. **Table 8** shows that the precision of VGG-16, ResNet-50, and ViT is 94.0%, 95.5%, and 96.2%, respectively. In these deep learning models, ViT has the highest precision, but it is still lower than the TLF method. The precision of the TLF method is 2.32% higher than that of the VGG-16 network. Compared with ResNet-50, the precision of the TLF method is improved by 0.82%. **Table 9** shows that the recall of VGG-16, ResNet-50, and ViT is 93.6%, 95.2%, and 96.0%, respectively. Compared with VGG-16 and ResNet-50, the recall of ViT is the highest. However, the recall of the TLF method is 0.32% higher than that of ViT. **Table 10** shows that the classification accuracy of VGG-16, ResNet-50, and ViT is 93.6%, 95.2%, and 96%, respectively. Compared with these deep learning models, the TLF method has higher accuracy. Although these deep learning models have achieved good performance on many datasets, the TLF method achieves better performance on the CW dataset.

**Table 8:** Precision of different deep learning models compared with the proposed methods.

Precision Method	Brick	Concrete	Foam	Wood	Plastic	Average
VGG-16 [9]	91.0%	100%	100%	100%	79.0%	94.0%
ResNet-50 [6]	100%	96.0%	89.3%	92.3%	100%	95.5%
ViT [8]	100%	100%	96.0%	92.0%	93.0%	96.2%
FLF	96. 7%	93.5%	93.8%	93.6%	98.4%	95.2%
TLF	97.6%	93.6%	96.8%	94.4%	99.2%	**96.3%**

**Table 9:** Recall of different deep learning models compared with the proposed methods.

Recall Method	Brick	Concrete	Foam	Wood	Plastic	Average
VGG-16 [9]	84.0%	92.0%	100%	92.0%	100%	93.6%
ResNet-50 [6]	92.0%	96.0%	100%	96.0%	92.0%	95.2%
ViT [8]	92.0%	96.0%	100%	92.0%	100%	96.0%
FLF	94.4%	92.0%	96.0%	94.4%	99.2%	95.2%
TLF	98.4%	93.6%	96.0%	94.4%	99.2%	**96.3%**

**Table 10:** Accuracy of different deep learning models compared with the proposed methods.

	VGG-16 [9]	ResNet-50 [6]	ViT [8]	FLF	TLF
Accuracy	93.6%	95.2%	96.0%	95.2%	**96.3%**

## 4. Conclusions

With the increasing focus on preserving the environment, CW recycling has become an important topic. Sorting a large amount of CW precisely and quickly is an urgent problem. This research shows that it is feasible to classify CW by computer vision. Motivated by the characteristics of the human visual system, the TLF method is proposed to classify CW materials in this work. The TLF method is based on a joint model of feature-level fusion and decision-level fusion. For the former, a statistical histogram and a BoVW method are applied to capture color features and HOG descriptors from a CW image, respectively. Moreover, inspired by feature-level fusion, a new feature named Color-HOG is constructed. For the latter, we fuse decisions from two base classifiers, which are learned from HH features and Color-HOG features. We name the model based on feature-level fusion as FLF, which is a special case of the TLF method. Compared with other state-of-the-art methods, the FLF method and TLF method have higher accuracy. The classification accuracy of the FLF method based on three base classifiers is 95.2%, 94.4%, and 92.96%, which is higher than that of the other state-of-the-art methods. Experiments demonstrate that Color-HOG is a robust feature for representing the discriminative characteristics of CW. Compared with the FLF method, the TLF method has higher accuracy: the accuracy of the TLF method based on the SVM classifier is 1.12% higher than that of the FLF method. In addition, we conduct experiments under various conditions. The experimental results also show that the proposed method has excellent performance under different conditions. In other words, the TLF method is an effective tool to promote the sorting and recycling of CW. This will be beneficial to reducing construction and CW management costs.

## Supporting information

S1 File (DOCX)
