# Supplementary material for: A new method of construction waste classification based on two-level fusion
Source: PLoS One. 2022 Dec 27;17(12):e0279472. doi: 10.1371/journal.pone.0279472 (PMC9794073; doi:10.1371/journal.pone.0279472)
Supplement: S1 File — (DOCX) [file pone.0279472.s001.docx]

Relevant data are available at https://github.com/zhaohuixuan/FLF.git
